# Can Evoked Potential Changes during the Superficial Temporal Artery-Middle Cerebral Artery Bypass Surgery Predict Postoperative Improvement of Cerebral Perfusion and Functional Status?

**DOI:** 10.3390/brainsci11111478

**Published:** 2021-11-08

**Authors:** Dougho Park, Suntak Jin, Youngsoo Kim, Yeon-Ju Choi, Daeyoung Hong, Byung Hee Kim, Sang-Eok Lee, Kwansang Cho, Ji Kang Park, Mun-Chul Kim

**Affiliations:** 1 Department of Rehabilitation Medicine, Pohang Stroke and Spine Hospital, Pohang 37659, Korea; parkdougho@gmail.com (D.P.); higpf@naver.com (B.H.K.); neopyte75@hotmail.com (S.-E.L.); 2 Department of Neurosurgery, Pohang Stroke and Spine Hospital, Pohang 37659, Korea; zin614@gmail.com (S.J.); Youngsooooo@gmail.com (Y.K.); ns_yeonju@naver.com (Y.-J.C.); hongdy2000@gmail.com (D.H.); 3 Department of Anesthesiology, Pohang Stroke and Spine Hospital, Pohang 37659, Korea; kscho1120@gmail.com; 4 Department of Radiology, Pohang Stroke and Spine Hospital, Pohang 37659, Korea; jkcontrast@gmail.com

**Keywords:** cerebral infarction, cerebral revascularization, intraoperative neurophysiological monitoring, motor evoked potential, somatosensory evoked potential, perfusion weighted image

## Abstract

Background: We investigated evoked potential (EP) changes during superficial temporal artery to middle cerebral artery (STA-MCA) bypass surgery and their correlations with imaging and clinical findings postoperatively. Methods: This retrospective study included patients who underwent STA-MCA bypass surgery due to ischemic stroke with large artery occlusion (MB group). Patients who underwent unruptured MCA aneurysm clipping were enrolled in the control group (MC group). Median and tibial somatosensory evoked potentials (SSEP), and motor evoked potentials recorded from the abductor pollicis brevis (APB-MEP) and abductor hallucis (AH-MEP) were measured intraoperatively. Modified Rankin scale (mRS) and perfusion-weighted imaging (PWI) related variables, i.e., mean transit time (MTT) and time to peak (TTP), were assessed. Results: Δmedian SSEP, ΔAPB-MEP, and ΔAH-MEP were significantly higher in the MB group than in the MC group (*p* = 0.027, *p* = 0.006, and *p* = 0.015, respectively). APB-MEP and AH-MEP amplitudes were significantly increased at the final measurement (*p* = 0.010 and *p* < 0.001, respectively). The ΔTTP asymmetry index was moderately correlated with ΔAPB-MEP (*r* = 0.573, *p* = 0.005) and ΔAH-MEP (*r* = 0.617, *p* = 0.002). ΔAPB-MEP was moderately correlated with ΔMTT (*r* = 0.429, *p* = 0.047) and ΔmRS at 1 month (*r* = 0.514, *p* = 0.015). Conclusions: MEP improvement during STA-MCA bypass surgery was partially correlated with PWI and mRS and could reflect the recovery in cerebral perfusion.

## 1. Introduction

Superficial temporal artery to middle cerebral artery (STA-MCA) bypass is a surgical treatment that involves extracranial to intracranial revascularization [1]. It is mainly performed for diseases such as ischemic stroke secondary to moyamoya disease, intracranial giant aneurysm, and intracranial tumor resection requiring vessel sacrifice [2,3,4]. In patients with cerebral ischemia due to large artery occlusions, STA-MCA bypass surgery can be performed to compensate for the loss of cerebral perfusion on the side of the lesion, if early thrombolysis or endovascular recanalization fails or is ineffective [5].

The representative methods of directly evaluating vascular patency during STA-MCA bypass surgery are microvascular Doppler ultrasonography and indocyanine green angiography [6,7]. The advantages of these flow-oriented evaluations are that they can confirm real-time blood flow intraoperatively, making them essential tools in STA-MCA bypass surgery [8]. Perfusion-weighted magnetic resonance imaging (PWI) performed before and after surgery is a good technique for the quantitative evaluation of regional cerebral blood flow. Previous studies have compared PWI findings before and after STA-MCA bypass surgery, and most of them have consistently reported its ability in demonstrating better cerebral perfusion after surgery [9,10,11]. 

Intraoperative neurophysiological monitoring (IONM) is widely used in open cranial surgeries, mainly as a precautionary measure to detect adverse events during surgery and to minimize the neural insult through subsequent rescue interventions [12,13]. Many previous studies have demonstrated its efficacy in reducing instances of postoperative neurologic deficits (PND) and in the attainment of better outcomes with open cranial surgeries [14,15,16].

Meanwhile, the role of IONM in predicting the patient’s postoperative recovery has also recently attracted attention, particularly in cervical decompression surgeries [17,18] and cerebral endovascular recanalization [19]. However, until now, few studies have been done on its ability to predict recovery post open cranial surgeries. IONM has a distinct advantage over PWI given to its ability to identify neurophysiological changes intraoperatively, through changes in evoked potentials (EP) [20]. Similarly, IONM can also elucidate the neurological functional status, while flow-oriented methods cannot [21].

This study aimed to confirm whether EP measured by IONM during STA-MCA bypass surgery could significantly be improved after vascular anastomosis. We also compared PWI findings with functional changes before and after surgery. Finally, we attempted to assess whether EP changes during surgery were associated with postoperative PWI changes and functional outcomes.

## 2. Materials and Methods

### 2.1. Patient Inclusion and Clinical Assessment

This was a single-center, retrospective study, with the sampling period extending from March 2017 to June 2020. This study was reviewed and approved by the institutional review board of Pohang Stroke and Spine hospital (approval number: PSSH0475-202102-HR-010-01). The requirement for informed consent was waived due to the retrospective nature of this study. All procedures performed in the study were conducted according to the guidelines of the Declaration of Helsinki.

**Figure 1:**
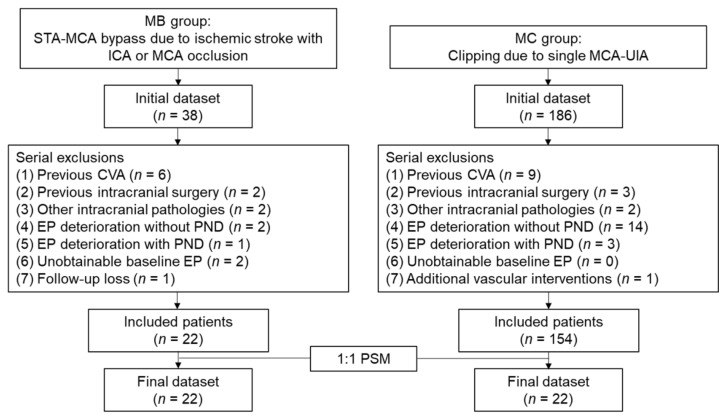
Flowchart of patient enrollment. MB, middle cerebral artery bypass surgery; MC, middle cerebral artery clipping surgery; STA, superficial temporal artery; MCA, middle cerebral artery; ICA, internal carotid artery; EP, evoked potential; PND, postoperative neurologic deficit; PSM, propensity score matching.

We enrolled patients who underwent STA-MCA bypass surgery due to ischemic stroke with unilateral internal carotid artery (ICA) or MCA occlusion and designated them as the STA-MCA bypass surgery group (MB group). During the same sampling period, patients who underwent single unruptured intracranial aneurysm clipping of the MCA with IONM were enrolled in the control group (MC group). In both groups, the patients with the following characteristics were excluded: (1) previous cerebrovascular accident or intracranial surgical history; (2) concomitant intracranial pathologies such as moyamoya disease, infection, tumor, or vascular malformation; (3) intraoperative EP deterioration due to adverse surgical event; (4) occurrence of a newly developed PND; or (5) intraoperatively unobtainable EP. Additionally, in the MB group, patients who were not followed up at 1- or 6-months after the surgery were also excluded. In the MC group, patients who simultaneously underwent additional treatment procedures such as bypass surgery or endovascular coiling were also excluded. Finally, in the MB group, 22 patients were enrolled, with an average age of 65.2 ± 10.4 years and comprising 9 (40.9%) male patients. In the MC group, 154 patients were enrolled with an average age of 61.5 ± 8.9 years and consisting of 41 (26.6%) male patients. There were no significant differences between the groups in terms of age and sex. The flowchart depicting patient enrollment is shown in Figure 1. 

We assessed patients for vascular risk factors such as hypertension, diabetes, hyperlipidemia, cardiac problems (coronary artery disease or symptomatic arrhythmia), and smoking. The functional status of patients in the MB group was measured by the modified Rankin scale (mRS) preoperatively, at 1 month, and at 6 months postoperatively. These measurements were double-checked for each patient by experienced neurosurgeons and rehabilitation specialists. The difference between the preoperative values of mRS and the postoperative values at 1-month and 6-months was defined as delta (Δ) mRS at 1 month and ΔmRS at 6 months, respectively. 

### 2.2. Surgical Procedures and Anesthesia 

For STA dissection, we would typically begin mapping the STA from the bifurcation of the frontal and parietal branches using a handheld Doppler. Generally, the parietal branch of the STA would be harvested if it was found to be suitable for anastomosis by preoperative angiography. If not, we would use the frontal branch of the STA instead. Then, a curvilinear incision would be planned over the STA, and soft-tissue dissection would be performed. After sufficient length of the donor STA was secured, it would be tied and cut.

**Figure 2:**
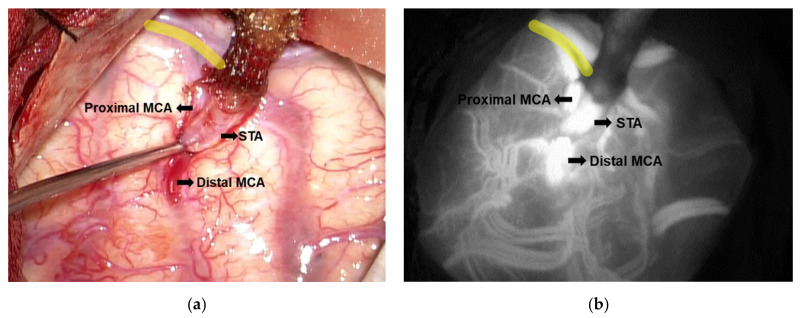
Anastomosis site of the superficial temporal artery- middle cerebral artery (STA-MCA) bypass. (**a**) The gross appearance shows the completed micro-anastomosis between STA and MCA. (**b**) The patency of the anastomosis site as confirmed by indocyanine green angiography. Yellow lines indicate the sylvian fissure. STA, superficial temporal artery; MCA, middle cerebral artery.

A small craniotomy would then be performed over the frontotemporal region. We would locate an M4 branch of the MCA emerging from the Sylvian fissure, preferentially over 1.0 mm in cross-sectional width and perpendicular to the Sylvian fissure, if possible. An end-to-side micro-anastomosis would then be performed with the use of 10-0 Monosof™ suture (Medtronic, Minneapolis, MN, USA) (Figure 2a). Finally, patency of the bypass would be confirmed using microvascular Doppler ultrasonography and indocyanine green angiography (Figure 2b and Supplementary Video S1).

Total intravenous anesthesia was used for all included surgeries. Propofol (3–5 mg/mL) and remifentanil (3–5 ng/mL) would be applied for induction, and a continuous infusion of propofol (2.5–3.5 mg/mL) and remifentanil (2.5–4.5 mg/mL) for maintenance. The bispectral index ranged from 30 to 60. No inhalation anesthetics were administered during the surgery. A single bolus of a neuromuscular blocking agent (rocuronium bromide, 0.4–0.5 mg/kg) would be administered before intubation. There was no continuous infusion during surgery.

### 2.3. Intraoperative Neurophysiological Monitoring Protocol

IONM procedures were conducted using XLTEK Protektor 32 (Natus Medical Inc., Oakville, ON, Canada). IONM modalities such as motor evoked potential (MEP), somatosensory evoked potential (SSEP), and electroencephalogram were used for open cranial surgeries.

To evoke MEP, transcranial electrical stimulation with subdermal needle electrodes was applied to C1 and C2 following the International 10–20 system. The stimulation methods were as follows: a repeatable train of five pulses with a pulse duration of 0.05 ms, interstimulus interval of 1–4 ms, and stimulation strength of 200–350 V. When the stimulation intensity was determined from the baseline EP, we maintained the constant intensity until the operation was completed. The filter range was set to 10–3000 Hz. MEP recordings were performed using the belly tendon method in the abductor pollicis brevis (APB-MEP) and abductor hallucis (AH-MEP) muscles. 

SSEP stimulation was performed at 1.75 Hz with 0.3 ms duration square-wave pulses. The stimulation intensity was 25 mA for the median SSEP and 30 mA for the tibial SSEP. The SSEP recording electrodes were located at C3’, C4’, Cz, and C5 according to the International 10–20 system, and the reference electrode was located at FPz. The filter range was set to the 30–1000 Hz range.

**Figure 3:**
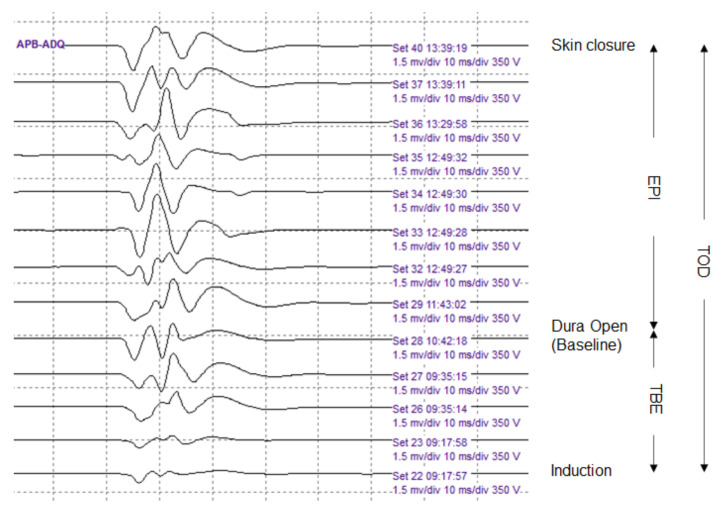
Serial abductor pollicis brevis motor evoked potentials (APB-MEPs) during the surgery. The APB-MEPs show a gradual increase in amplitude. We defined three time-related covariates. The time to baseline EP (TBE) was designated as the time from induction to baseline EP measurement. The EP interval (EPI) represented the time from baseline EP to final EP measurements. Finally, the total operation duration (TOD) was the time from induction to final EP measurement.

The baseline EP was obtained just before dura opening, and the final EP was defined as the EP obtained immediately after skin closure. The amplitude of each EP modality—APB-MEP, AH-MEP, median SSEP, and tibial SSEP, was used as a variable on the side of the lesion, and the unit of measurement was µV. Time to baseline EP (TBE), EP interval (EPI), and total operation duration (TOD) were measured as time-related covariates. TBE was defined as the time from induction to baseline EP measurement, and EPI was defined as the time from baseline EP to final EP measurements. TOD was defined as the time from induction to the final EP measurement (Figure 3). We defined Δ (%) EP as the change in the final EP amplitude, expressed as a percentage of the baseline EP amplitude, represented by the following equation:

ΔEP (%) = ((Final EP amplitude − Baseline EP amplitude)/Baseline EP amplitude) × 100

### 2.4. Perfusion-Weighted Imaging Protocol 

**Figure 4:**
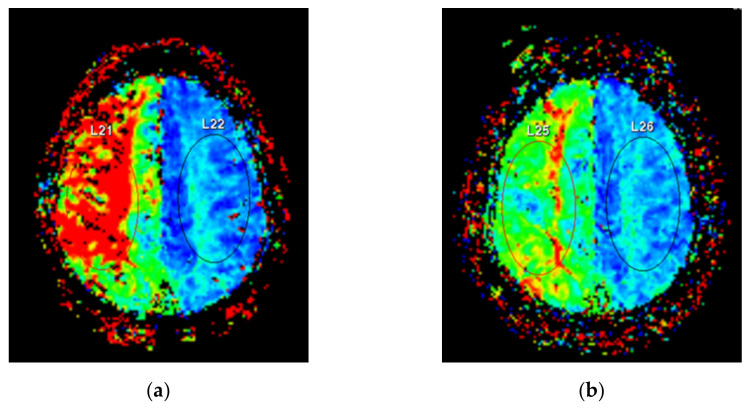
Perfusion weighted magnetic resonance imaging. Regions of interest were located at bilateral centrum semiovale areas. (**a**) Preoperative imaging reveals delayed time to peak at the right subcortical area. (**b**) Postoperative imaging shows improved cerebral perfusion with decreased color difference between the affected and unaffected sides.

We used a 3.0-tesla whole-body magnetic resonance system (Ingenia 3.0T CXQ, Phillips, Eindhoven, Netherlands). PWI was performed using the following protocol: TR = 1300 ms; TE = 30 ms, flip angle = 30°, matrix = 128 × 128, field of view = 240 × 240 mm, number of signals averaged = 1, and slice thickness = 5 mm. Gadobutrol (Gadovist^®^ 1.0, Bayer Schering Pharma, Berlin, Germany) was used as a contrast agent, and 10 mL was injected intravenously at a rate of 2.5 mL/s. Regions of interest (ROIs) were established in the bilateral centrum semiovale area. The same ROI location was selected for all measurements in each patient (Figure 4). 

All patients in the MB group underwent PWI preoperatively and 1 week after surgery. We used the mean transit time (MTT) and time-to-peak (TTP) as the PWI-related variables for the affected side. We also defined the asymmetry index (AI) reported by Bozzao et al. [10] as a PWI-related variable, defined as the PWI parameter measured on the affected side divided by that on the unaffected side. Further, we calculated MTT AI and TTP AI. ΔMTT AI was defined as the change in MTT AI postoperatively, divided by the preoperative MTT AI, and was expressed as a percentage. ΔTTP AI was defined in a similar manner. The related equations are as follows:

ΔMTT = Preoperative MTT − Postoperative MTT ΔTTP = Preoperative TTP − Postoperative TTP MTT AI = affected side MTT/unaffected side MTT TTP AI = affected side TTP/unaffected side TTP ΔMTT AI = ((Preoperative MTT AI − Postoperative MTT AI)/Preoperative MTT AI) × 100 ΔTTP AI = ((Preoperative TTP AI − Postoperative TTP AI)/Preoperative TTP AI) × 100

### 2.5. Statistical Analysis

All statistical analyses were performed using R software 4.0.3 provided by R Core Team (R Foundation for Statistical Computing, Vienna, Austria). Statistical significance was set at *p* < 0.05. The Shapiro–Wilk normality test was conducted for all continuous variables. Parametric data were expressed as the mean ± standard deviation and non-parametric data as median values (interquartile range). A two-sample *t*-test was used to compare parametric data, and the Wilcoxon rank-sum test with continuity correction was used to compare non-parametric data. A paired *t*-test or Wilcoxon matched-pairs signed-rank test was applied to compare baseline and final EP in the same patient and PWI parameters before and after surgery. Categorical variables were expressed as frequency and proportion, and the chi-squared (trend) test or Fisher exact test was applied to test for associations. Spearman’s rank correction test was used to determine the correlation between changes in EP and changes in other variables. Finally, linear regression analysis was performed to test EP as a potential predictor of PWI changes.

We performed propensity score matching (PSM) using the “MatchIt” package of R software [22]. The following covariates were used to conduct logistic regression: age, sex, vascular risk factors, operation side, and time-related variables (TOD, TBE, and EPI). Patients were matched using the nearest-neighbor method with no replacement and a 1:1 matching. 

## 3. Results

### 3.1. Baseline Characteristics and Propensity Score Matching

The MC group included more right-sided surgeries than the MB group (99 (64.3%) and 8 (36.4%), respectively; *p* = 0.023). As for vascular risk factors, hypertension and diabetes were observed in 86.4% and 54.4% of patients in the MB group, respectively, significantly higher than that observed in the MC group (51.9% and 11.0%; *p* = 0.005 and *p* < 0.001, respectively). On the other hand, the rate of hyperlipidemia was higher in the MC group (21.4%) than in the MB group (9.1%), though the difference was not statistically significant. Although instances of cardiac problems and smoking were higher in the MB group, no statistical significance was observed between the two groups. The median time from stroke onset to surgery was 2 months (0.0, 5.2) in the MB group.

The TOD of the MB group was 273.4 ± 48.8 min, significantly longer than the 210.1 ± 54.5 min of the MC group (*p* < 0.001). The TBE of the MB group was 85.0 min (73.0, 99.0), also significantly longer than the 57.0 min (47.0, 64.0) of the MC group (*p* < 0.001). Similarly, EPI was also significantly longer in the MB group than in the MC group (183.7 ± 34.4 min and 153.3 ± 49.5 min, respectively; *p* < 0.001).

We conducted a comparative analysis of the MB and MC groups with 22 matched patients in each group after PSM. The TBE of the matched MC group was 69.0 min (60.0, 85.0), which was longer than that of the total MC group. However, it was still significantly shorter than that of the MB group (*p* = 0.018). Other covariates were not significantly different between the two groups after PSM. 

The baseline characteristics and comparative analyses of the total MC, matched MC, and MB groups are summarized in Table 1. The stroke-related factors and functional outcomes are summarized in Supplementary Table S1. 

### 3.2. Comparison of EP Changes between the MB and MC Group

The Δmedian SSEP was 13.8% (0.6, 41.3) in the MB group, reduced to −4.3% (−19.8, 11.7) in the MC group (*p* = 0.027). The Δtibial SSEP was not significantly different between the MB and MC groups (9.3 ± 26.8% and 1.5 ± 23.7%, respectively; *p* = 0.318). The ΔAPB-MEP of the MB group was 20.7% (5.6, 71.6), significantly greater than the 2.5% (−14.5, 12.9) of the MC group (*p* = 0.006). The ΔAH-MEP was also significantly higher in the MB group [29.2% (8.6, 55.2)] than in the MC group [9.7% (−5.2, 28.2); *p* = 0.015] (Table 2). 

### 3.3. Changes in Examined Parameters and Their Correlations in the MB Group

**Figure 5:**
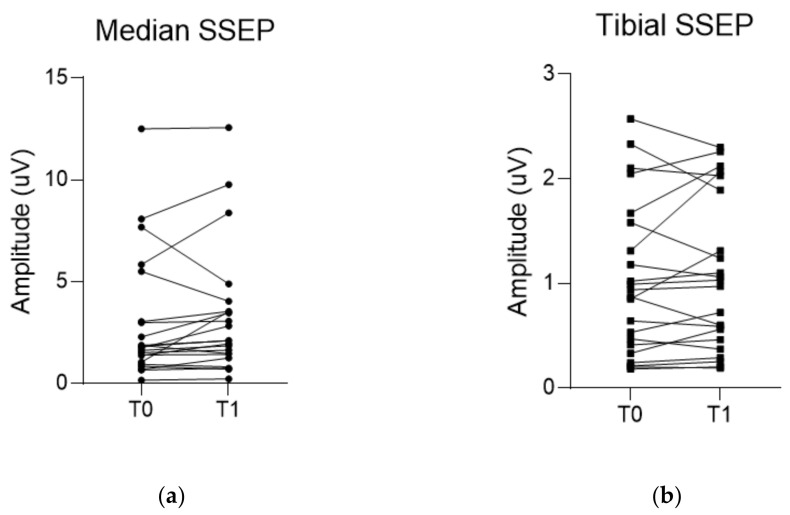

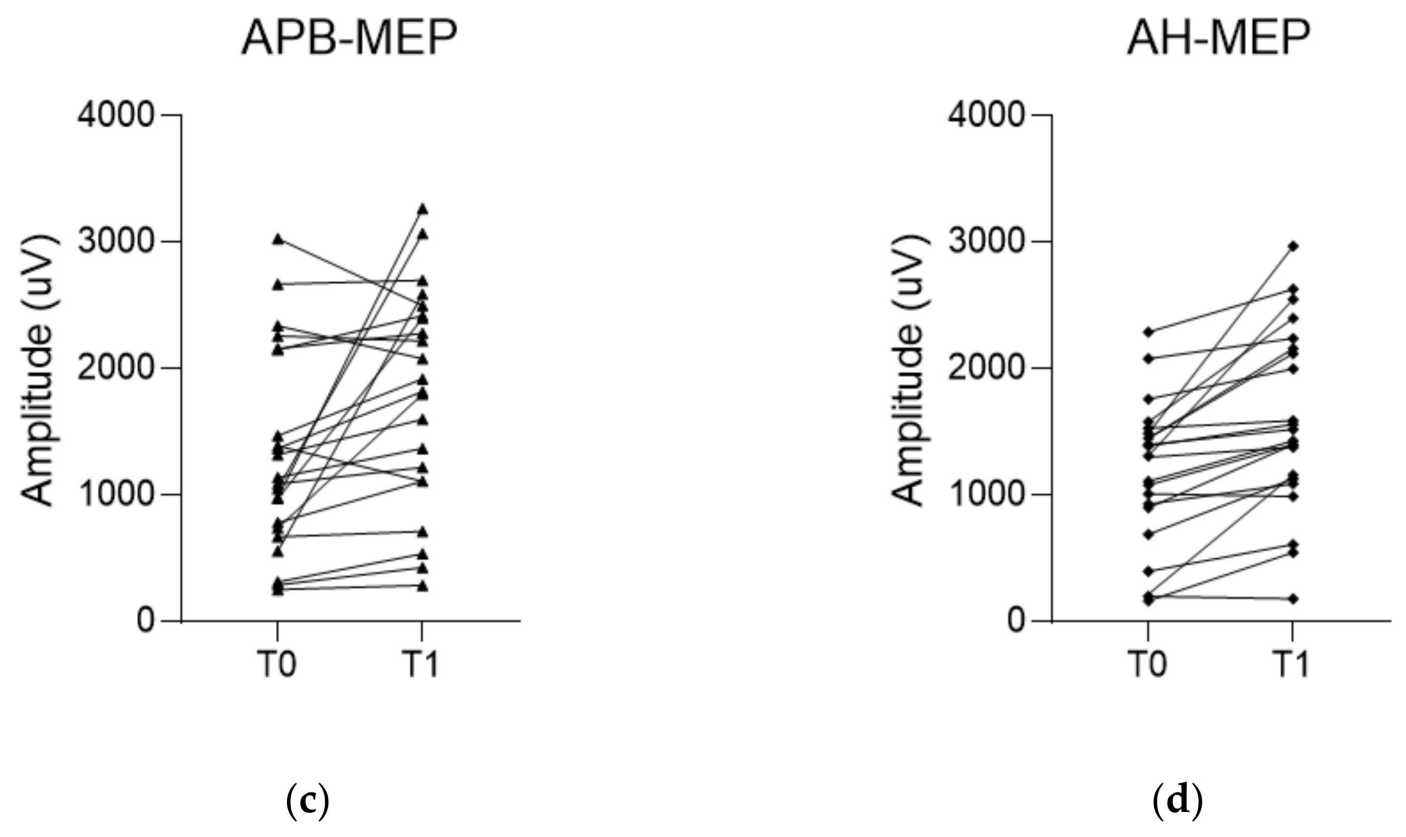
Changes in amplitude of each evoked potential (EP) during the surgery. (**a**,**b**) Median and tibial somatosensory evoked potential (SSEP) do not show a significant increase in amplitudes between baseline and final values. On the other hand, (**c**,**d**) motor evoked potentials recorded in the abductor pollicis muscle (APB-MEP) and abductor hallucis muscle (AH-MEP) show a significant increase in their amplitudes between baseline and final values. T0 indicates the baseline EP. T1 indicates the final EP.

The median SSEP showed a slight increase in amplitude from 1.8 µV (1.0, 3.0) to 2.1 µV (1.4, 3.5), though it was not statistically significant (*p* = 0.051). Tibial SSEP showed little change from baseline amplitude of 0.9 µV (0.4, 1.6) to 1.0 µV (0.5, 1.9) to the final amplitude (*p* = 0.604). Meanwhile, the APB-MEP amplitude significantly increased from baseline 1318.9 ± 796.1 µV to the final value 1793.2 ± 856.0 µV (*p* = 0.010). Similarly, AH-MEP amplitude also significantly increased from a baseline of 1169.9 ± 576.2 µV to a final value of 1593.8 ± 721.6 µV (*p* < 0.001) (Table 3 and Figure 5).

For the PWI parameters, MTT significantly decreased from 12.4 seconds (10.1, 14.1) to 10.7 seconds (9.7, 12.6) (*p* = 0.026) and TTP significantly decreased from 32.4 ± 6.3 seconds to 29.8 ± 5.8 seconds (*p* = 0.012) after the surgery. Likewise, MTT AI also significantly decreased from 1.2 (1.1, 1.5) to 1.1 (1.1, 1.2) (*p* = 0.010). No difference in the median values of TTP AI were witnessed [1.1 (1.1, 1.2) to 1.1 (1.0, 1.1)], but overall, the postoperative values were significantly reduced (*p* < 0.001) (Table 3).

As for preoperative mRS, the highest grade was Grade 4 seen in 10 patients (45.5%), followed by Grade 3 in seven patients (31.8%). Postoperatively, mRS Grade 1 was the highest grade seen in nine patients (40.9%), followed by Grade 2 in six (27.3%); the distribution of mRS grades before and after the surgery showed significant differences (*p* < 0.001) (Table 3).

On correlation analyses between EP findings and other parameters, ΔSSEPs were not significantly associated with PWI parameters and mRS changes. Meanwhile, there were significant correlations between ΔTTP AI and ΔMEPs; a moderate correlation was found in ΔAPB-MEP (*r* = 0.573, *p* = 0.005) and ΔAH-MEP (*r* = 0.617, *p* = 0.002). ΔAPB-MEP also showed a moderate correlation with ΔMTT (*r* = 0.429, *p* = 0.047) and ΔmRS at 1 month (*r* = 0.514, *p* = 0.015). No other significant correlations between EP and mRS changes were identified (Table 4). Simple regression analyses between changes in EP and PWI parameters did not show any significant association (Table 5).

## 4. Discussion

In this study, we aimed to identify whether improved cerebral perfusion during STA-MCA bypass surgery was reflected by EP changes intraoperatively. To the best of our knowledge, this is the first systematic study to evaluate postoperative recovery potential after open cranial surgery with IONM. Therefore, our results can serve as a reference for future related research.

We noted that improvement in EP amplitude was significantly greater in the MB group than in the MC group. In line with the case-control study design, we tried to find an open cranial surgery group with minimal hemodynamic abnormality as a control group. Additionally, as with the STA-MCA bypass, we believed that a patient group who underwent unilateral MCA surgery was suitable. To satisfy these conditions, we finally set a single MCA aneurysm clipping group as the control group. Consequently, we confirmed significant differences in EP change between these two groups.

However, this improvement did not show any significant predictive power in simple regression analyses with PWI parameters, even if they were partially correlated with some of the PWI and mRS findings. This can be attributed to the following reasons. In contrast to the postoperative PWI parameters and mRS evaluated at one week and one month after the surgery, respectively, EP changes were evaluated intraoperatively. Therefore, it was presumed that the time for improved cerebral perfusion to be reflected after STA-MCA anastomosis was insufficient. In addition, a small number of MB group patients might have caused the regression analysis to fall short of statistical significance.

Studies have reported that changes in EP during IONM, especially MEP, can predict postoperative functional outcomes. Greve et al. [19] demonstrated that MEP recovery during mechanical endovascular thrombectomy (MET) was associated with functional improvement and was more useful than modified thrombolysis in the cerebral infarction grading system. Shiban et al. [23] presented similar results, showing that the recoveries of MEP and SSEP were better predictors of functional improvement than successful reperfusion during MET in ischemic stroke patients. However, previous studies have not reported a consensus on the amount of EP change, which could be defined as significant improvement during surgery. Some researchers have defined significant EP improvement as a 50% or greater increase in amplitude, which is IONM’s warning criteria applied in reverse [24]. According to this standard, all EP modalities in our results did not show any significant increase in their amplitude. A large-scale study will be needed to establish positive outcome criteria in the revascularization surgery of IONM in the future, akin to IONM’s warning criteria.

Due to the lack of consistent EP improvement criteria, we endeavored to identify the associated changes in imaging and functional findings to confirm the significance of EP changes. We found that only MEP showed a significant correlation with PWI changes, while SSEP did not. ABP-MEP also showed a significant correlation with functional changes at 1-month. However, the degree of EP change was not related to PWI findings in the regression analyses. Therefore, we can infer that though the increase in EP amplitude during the surgery reflects some improvement in cerebral perfusion and functional status, it is also limited to predicting the postoperative changes in cerebral perfusion shown on PWI. The reason that the significance of EP change in our study was relatively insufficient compared to the aforementioned studies on MET might be due to differences in the viable neuronal tissue potential. In other words, MET in other studies was performed in the hyperacute stage; however, in our patients, the median time to surgery from the onset of stroke was 2 months. This can be attributed to the different time points for applying the MET or bypass treatment in ischemic stroke with large artery occlusion. STA-MCA bypass surgery for patients with large artery occlusion is usually considered in cases with ineffective acute endovascular recanalization or inadequate blood circulation compensation in the ischemic area [25,26]. This difference in the timing of surgery may have affected EP response after reperfusion.

As mentioned above, MEP showed a more pronounced change between baseline and final measurements than did SSEP. This suggests that MEP has greater sensitivity in reflecting changes in cerebral perfusion than SSEP. These results have been echoed several times in previous studies. Horiuchi et al. [27] reported that MEP in MCA aneurysm surgery could reflect blood flow insufficiency well; however, they also reported that SSEP was not reliable, especially in regions supplied by the MCA branches and lenticulostriate arteries. Neuloh et al. [28] stated that MEP was superior in detecting motor impairment compared to SSEP or intraoperative Doppler ultrasonography during intracranial aneurysm surgeries. Although they discussed the reliability of these methods in detecting decreased blood flow during intracranial surgeries, these studies indirectly support our findings that MEP could reflect blood flow changes better than SSEP.

The EP modalities applied to IONM differ slightly in their coverage area. MEP is more sensitive to subcortical ischemia; on the other hand, SSEP is more sensitive to cortical ischemia [13,29]. The recipient vessel in STA-MCA bypass surgery is the M4 branch. Therefore, after the anastomosis, it is presumed that the antegrade flow mainly supplies the cortical areas; whereas the retrograde flow mainly supplies the white matter along the M2 branch and the deep portion of the cerebral hemisphere, which is related to lateral lenticulostriate arteries [30,31]. These hemodynamic changes could be another reason why our results did not show a significant association between EP changes and PWI findings compared to previous studies. In the case of MET, the improvement in the antegrade flow after recanalization may reflect improvement in MEP in the subcortical region [19]. On the other hand, in the case of STA-MCA bypass surgery, some degree of subcortical perfusion depends on the retrograde flow improvement; therefore, MEP change can be relatively less evident than that in MET cases [30,32]. Moreover, our finding of MEP improvement being more pronounced than SSEP improvement suggests that the response to cerebral perfusion changes might depend on which EP modality is applied rather than regional factors.

One of the strengths of this study is that we tried to obtain detailed and comparable results interpretable in actual clinical field settings by adjusting time-related factors. Time-related factors play an important role when interpreting MEP during IONM. Therefore, we attempted to minimize bias by reducing the differences in time-related factors between the MB and MC groups through PSM. However, after PSM, though the differences narrowed between the groups, TBE was still significantly longer in the MB group, which was considered a reflection of the STA dissection time before dura opening [33]. Short TBE may imply that the effect of neuromuscular blocking agent administered before intubation cannot be completely excluded. Thus, it might have acted as a factor in the MC group and underestimated the baseline MEP amplitude [34]. Nevertheless, since the MEP amplitude change in the MB group was significantly larger, the possibility that TBE difference acted as a bias in the comparative analysis between the two groups was considered to be minimal, even if the matching was not complete. 

The definition of baseline EP as that obtained just before dura opening is also related to this time-related factor. Several previous studies have already used EP obtained just before dura opening as baseline values when testing for the reliability of MEP interpretation during IONM in open cranial surgery [35,36]. We used rocuronium for intubation, which has a duration of action of 30–60 min [37]. TBE of the MC and MB groups were 69 and 85 min, respectively. Therefore, we can guarantee that we had obtained baseline MEP data, excluding the effect of the neuromuscular blocking agent used during intubation.

Another consideration in terms of time is the anesthetic fade effect. This may affect the final EP and can underestimate the increase in MEP amplitude reflecting blood flow improvement [38]. Ugawa et al. [39] studied anesthetic fade effects on MEP in spinal deformity surgeries. Their results revealed a significant decrease in MEP amplitude (16%) in the upper extremity 5 h after the initial propofol infusion and a significant decrease in MEP amplitude (10%) in the lower extremity 4 h after the initial propofol infusion. In our study, the mean surgical time in the MB group was 273.4 ± 48.8 min, and we cannot completely exclude the anesthetic fade effect in AH-MEP as well as APB-MEP. Therefore, caution is needed when interpreting MEP, especially the possibility of underestimation of amplitude improvement.

We also intended to match the vascular risk factors that could affect EP results through PSM. In the MB group, the rates of hypertension and diabetes were significantly higher than those in the MC group. In addition, cardiac problems and smoking rates were higher in the MB group, although the difference was not statistically significant. There was no significant difference in vascular risk factors between the MC and MB groups after PSM. Therefore, our comparative analysis of EP results was reliable.

Another noteworthy factor in our research method was the use of TTP as a variable. Previous studies on PWI in extracranial to intracranial bypass surgery have mainly targeted large aneurysms or moyamoya disease. Related studies have generally utilized regional cerebral blood flow, cerebral blood volume, and MTT as variables. Our study differs from previous studies in that we only targeted patients with acute or subacute stroke secondary due to large-artery occlusion, not related to moyamoya disease. We thus hypothesized that TTP would better reflect cerebral perfusion mismatch in patients with stroke [40,41]. Indeed, in our results, ΔMTT AI showed no significant correlation with EP changes; however, ΔTTP AI showed significant correlations with MEPs, in line with our hypothesis. In addition, Chen et al. [42] performed CT perfusion scans before and after STA-MCA bypass surgery in patients with moyamoya disease. They revealed that MTT and TTP showed significant changes postoperatively, while cerebral blood volume and cerebral blood flow did not change significantly immediately after the surgery. This suggests that TTP and MTT are rather sensitive in reflecting early blood flow changes after surgery. Therefore, based on these results, TTP and MTT were used as variables in our study.

We designated bilateral centrum semiovale as the ROI for PWI parameters. Centrum semiovale is suitable for evaluating overall MCA flow because it is supplied mainly by long arteries and arterioles [32,43]. In addition, from previous studies, centrum semiovale and basal ganglia are known to be regions with high vulnerability to cerebral hypoperfusion [43]. Kluytmans et al. [44] reported that the perfusion delay of white matter was significantly greater than that of gray matter in patients with unilateral ICA occlusion. Yamauchi et al. [45] confirmed susceptibility of white matter to a reduction in perfusion by revealing a selective hematocrit decrease in the centum semiovale region in the chronic carotid artery occlusion group.

Our study has the following limitations. This was a retrospective study conducted on a small number of participants. We aimed to identify EP improvement derived from increased perfusion; therefore, we only included patients who underwent uneventful surgeries. We designed the study to investigate the association between the IONM findings and postoperative recovery in stroke patients. Therefore, both the MB and MC groups excluded patients with adverse intraoperative events or PNDs, which may have contributed to a selection bias. We used mRS as a clinical indicator, which could not sufficiently reflect various aspects of ischemic stroke symptoms, especially non-motor symptoms that mainly reflect cortical function. Furthermore, the ROI for the cortical area was not established owing to the poor measurement reliability for each patient. Although ROI on the centrum semiovale allowed for a broad confirmation of hemodynamic changes in the subcortical area in the MCA territory [32], the amount of cortical reperfusion and its association with EP changes could not be investigated. 

## 5. Conclusions

The intraoperative EP improvement in STA-MCA bypass surgery can reflect recovery in cerebral perfusion; however, its association with postoperative imaging and functional changes has not been sufficiently demonstrated. In the future, large-scale, systemic studies are needed to establish IOMN criteria to predict positive outcomes in open cranial surgeries.

## Figures and Tables

**Table 1:** Baseline characteristics of patients.

	MC Group				MB Group
	Total (*n* = 154)	*p*	PSM (*n* = 22)	*p*	(*n* = 22)
Age, years	61.5 ± 8.9	0.074	61.5 ± 10.1	0.233	65.2 ± 10.4
Sex, male (%)	41 (26.6)	0.255	9 (40.9)	>0.999	9 (40.9)
Side, right (%)	99 (64.3)	0.023	10 (45.5)	0.759	8 (36.4)
Vascular risk factors					
Hypertension, *n* (%)	80 (51.9)	0.005	18 (81.8)	>0.999	19 (86.4)
Diabetes, *n* (%)	17 (11.0)	<0.001	9 (40.9)	0.546	12 (54.5)
Hyperlipidemia, *n* (%)	33 (21.4)	0.255	4 (18.2)	0.664	2 (9.1)
Cardiac problems^a^, *n* (%)	10 (6.5)	0.211	3 (13.6)	>0.999	3 (13.6)
Smoking, *n* (%)	21 (13.6)	0.114	6 (27.3)	>0.999	6 (27.3)
Time-related factors					
TOD, min	210.1 ± 54.5	<0.001	242.7 ± 56.3	0.060	273.4 ± 48.8
TBE, min	57.0 (47.0, 64.0)	<0.001	69.0 (60.0, 85.0)	0.018	85.0 (73.0, 99.0)
EPI, min	153.3 ± 49.5	<0.001	170.8 ± 51.1	0.331	183.7 ± 34.4

MC, middle cerebral artery clipping surgery; MB, middle cerebral artery bypass surgery; PSM, propensity score matching; TOD, total operation duration; TBE, time to baseline evoked potential; EPI, evoked potentials’ time interval between baseline and final measurements.; ^a^ coronary artery disease or symptomatic arrhythmia.

**Table 2:** Changes in the amplitude of each evoked potential during surgery.

	MB Group (*n* = 22)	MC Group (*n* = 22)	*p*-Value
ΔMedian SSEP (%)	13.8 (0.6, 41.3)	−4.3 (−19.8, 11.7)	0.027
ΔTibial SSEP (%)	9.3 ± 26.8	1.5 ± 23.7	0.318
ΔAPB-MEP (%)	20.7 (5.6, 71.6)	2.5 (−14.5, 12.9)	0.006
ΔAH-MEP (%)	29.2 (8.6, 55.2)	9.7 (−5.2, 28.2)	0.015

MB, middle cerebral artery bypass surgery; MC, middle cerebral artery clipping surgery; SSEP, somatosensory evoked potential; APB, abductor pollicis brevis; MEP, motor evoked potential; AH, abductor hallucis.

**Table 3:** Changes in examined parameters in the MB group.

	T0 ^a^	T1 ^b^	*p*-Value
Median SSEP (µV)	1.8 (1.0, 3.0)	2.1 (1.4, 3.5)	0.051
Tibial SSEP (µV)	0.9 (0.4, 1.6)	1.0 (0.5, 1.9)	0.604
APB-MEP (µV)	1318.9 ± 796.1	1793.2 ± 856.0	0.010
AH-MEP (µV)	1169.9 ± 576.2	1593.8 ± 721.6	<0.001
MTT (s)	12.4 (10.1, 14.1)	10.7 (9.7, 12.6)	0.026
TTP (s)	32.4 ± 6.3	29.8 ± 5.8	0.012
MTT AI ^c^	1.2 (1.1, 1.5)	1.1 (1.1, 1.2)	0.010
TTP AI ^c^	1.1 (1.1, 1.2)	1.1 (1.0, 1.1)	<0.001
mRS, *n* (%)			<0.001
0	0 (0.0)	1 (4.5)	
1	0 (0.0)	9 (40.9)	
2	5 (22.7)	6 (27.3)	
3	7 (31.8)	3 (13.6)	
4	10 (45.5)	3 (13.6)	

MB, middle cerebral artery bypass surgery; T0, time point pre; T1, time point post; SSEP, somatosensory evoked potential; APB, abductor pollicis brevis; MEP, motor evoked potential; AH, abductor hallucis; MTT, mean transit time; TTP, time to peak; AI, asymmetry index; mRS, modified Rankin scale. ^a^ indicating baseline value for evoked potentials and preoperative examination for perfusion weighted imaging findings and mRS; ^b^ indicating final value for evoked potentials, postoperative 1 week’s perfusion weighted imaging findings, and postoperative 1 month’s mRS; ^c^ AI = affected side/unaffected side.

**Table 4:** Correlation coefficients (*p*-value) between EP changes and other examined parameters in the MB group.

	ΔMTT ^a^ (s)	ΔTTP ^a^ (s)	ΔMTT AI ^b^ (%)	ΔTTP AI ^b^ (%)	ΔmRS ^c^ at 1 M	ΔmRS ^c^ at 6 M
ΔMedian SSEP ^d^ (%)	0.102 (0.651)	0.194 (0.388)	−0.069 (0.759)	0.112 (0.619)	0.374 (0.087)	0.422 (0.050)
ΔTibial SSEP ^d^ (%)	−0.139 (0.536)	−0.108 (0.633)	−0.124 (0.584)	0.130 (0.563)	−0.060 (0.794)	−0.116 (0.608)
ΔAPB-MEP ^d^ (%)	0.429 (0.047)	0.043 (0.848)	0.348 (0.112)	0.573 (0.005)	0.514 (0.015)	0.271 (0.222)
ΔAH-MEP ^d^ (%)	0.415 (0.055)	0.325 (0.140)	0.344 (0.117)	0.617 (0.002)	0.332 (0.131)	0.183 (0.416)

EP, evoked potential; MB, middle cerebral artery bypass surgery; MTT, mean transit time; AI, asymmetry index; TTP, time to peak; M, month(s); mRS, modified Rankin scale; SSEP, somatosensory evoked potential; APB, abductor pollicis brevis; MEP, motor evoked potential; AH, abductor hallucis. ^a^ Preoperative − Postoperative; ^b^ ((Preoperative AI − Postoperative AI)/Preoperative AI) × 100; ^c^ Preoperative − Postoperative; ^d^ ((Final EP amplitude − Baseline EP amplitude)/Baseline EP amplitude) × 100.

**Table 5:** Univariate analysis of simple linear regression model in the MB group.

	ΔMTT ^a^ (s)		ΔTTP ^a^ (s)		ΔMTT AI ^b^ (%)		ΔTTP AI ^b^ (%)	
	*β* ± SE	*p*	*β* ± SE	*p*	*β* ± SE	*p*	*β* ± SE	*p*
ΔMedian SSEP^c^ (%)	−0.080 ± 0.083	0.346	0.007 ± 0.051	0.890	−0.032 ± 0.063	0.618	−0.156 ± 0.026	0.562
ΔTibial SSEP ^c^ (%)	−0.164 ± 0.173	0.355	−0.072 ± 0.106	0.508	−0.082 ± 0.131	0.533	−0.014 ± 0.056	0.808
ΔAPB-MEP ^c^ (%)	0.021 ± 0.049	0.667	0.001 ± 0.030	0.975	0.025 ± 0.036	0.505	0.010 ± 0.015	0.532
ΔAH-MEP ^c^ (%)	0.034 ± 0.048	0.487	0.019 ± 0.029	0.532	0.013 ± 0.036	0.727	0.012 ± 0.015	0.449

MB, middle cerebral artery bypass surgery; MTT, mean transit time; TTP, time to peak; AI, asymmetry index; SE, standard error; SSEP, somatosensory evoked potential; APB, abductor pollicis brevis; MEP, motor evoked potential; AH, abductor hallucis. ^a^ Preoperative − Postoperative; ^b^ ((Preoperative AI − Postoperative AI)/Preoperative AI) × 100; ^c^ ((Final EP amplitude − Baseline EP amplitude)/Baseline EP amplitude) × 100.

## Data Availability

The datasets analyzed during the current study are available from the corresponding author on reasonable request.

## Supplementary Materials

The following are available online at https://www.mdpi.com/article/10.3390/brainsci11111478/s1, Video S1: The patency of anastomosis site is confirmed using indocyanine green angiography. Table S1: Patients and their stroke-related factors in the MB-group.
