# Supplementary material for: Can Evoked Potential Changes during the Superficial Temporal Artery-Middle Cerebral Artery Bypass Surgery Predict Postoperative Improvement of Cerebral Perfusion and Functional Status?
Source: Brain Sci. 2021 Nov 8;11(11):1478. doi: 10.3390/brainsci11111478 (PMC8615961; doi:10.3390/brainsci11111478)
Supplement: Supplementary file 1 [file brainsci-11-01478-s001.zip › Supplementary table.pdf]

Table S1. Patients and their stroke related factors in the MB-group.

Cases	Age	Sex	Side	Angiographic findings	Lesion territory	Infraction volume[a] (mm^3)	Onset to Bypass (months)	PreOP-mRS	mRS at 1m	mRS at 6m
1	76	Female	Right	ICA occlusion	MCA superior division	13	1	2	1	1
2	56	Male	Right	Distal ICA multiple thrombus and severe stenosis	MCA total	54.8	0	3	1	1
3	55	Male	Left	Distal M1 severe stenosis	MCA inferior division	5.1	5	3	1	0
4	69	Male	Right	M1 occlusion	MCA perforating branches	8.4	4	3	1	0
5	61	Female	Right	M1 occlusion	MCA perforating branches	11.8	12	2	0	0
6	55	Female	Right	ICA total occlusion	MCA perforating branches	13.2	0	4	3	1
7	79	Female	Left	M1 occlusion	MCA inferior division	12.8	2	3	2	1
8	56	Female	Right	M2 occlusion	MCA superior division	7.2	3	2	2	1
9	57	Male	Left	distal M1 occlusion	MCA superior division	9.2	0	4	1	1
10	42	Male	Left	ICA occlusion	MCA total	52.8	12	3	1	0
11	51	Female	Right	M1 occlusion	MCA perforating branches	8.2	6	4	2	2
12	75	Female	Left	M2 occlusion	MCA superior division	7.8	0	4	2	1
13	63	Male	Right	proximal ICA occlusion	MCA borderzone areas	10.1	0	2	1	0
14	69	Female	Left	ICA occlusion	MCA borderzone areas	3.8	1	4	3	3
15	75	Male	Left	ICA occlusion	MCA superior division	2.7	12	4	4	3
16	68	Female	Left	ICA occlusion	MCA superior division	16.9	1	3	1	0
17	75	Female	Left	M1 severe stenosis	MCA perforating branches	11.5	11	4	4	4
18	66	Female	Left	ICA severe stenosis	MCA borderzone areas	2.3	3	4	3	2
19	82	Female	Left	ICA severe stenosis	MCA inferior division	36.4	0	4	2	1
20	73	Male	Left	ICA occlusion	MCA total	21.5	0	4	4	3

21	60	Female	Left	M1 occlusion	MCA borderzone areas	7.4	2	3	2	1
22	72	Male	Left	M1 occlusion	MCA inferior division	2.1	2	2	1	0

OP, operation; mRS, modified Rankin scale; m, month(s); ICA, internal carotid artery; MCA, middle cerebral artery.

[a]Jung et al.'s method [1]; the sum of the infarct area on each DWI slice (slice thickness + interslice gap).

Reference

1. Jung, J.M.; Kwon, S.U.; Lee, J.H.; Kang, D.W. Difference in infarct volume and patterns between cardioembolism and internal carotid artery disease: focus on the degree of cardioembolic risk and carotid stenosis. *Cerebrovascular Dis* **2010**, 29, 490-496, doi: 10.1159/000297965.
